# YBX1 orchestrates LDHA-mediated metabolic reprogramming and NF-κB activation to drive clear cell renal cell carcinoma progression

**DOI:** 10.1038/s41419-025-08261-0

**Published:** 2026-01-08

**Authors:** Yihan Dong, Aixin Qiu, Shuang Liu, Yuejing Pan, Tianyu Lin, Rui Wang, Ruibing Chen, Huamao Jiang, Yang Yu, Yong Wang, Dan Yue

**Affiliations:** 1 Division of Medical Technology, Tianjin Medical University, Tianjin, China; 2 Department of Urology, The Second Hospital of Dalian Medical University, Dalian, China; 3 Department of Clinical Laboratory, Institute of Translational Medicine, Renmin Hospital of Wuhan University, Wuhan, Hubei China; 4 Department of Urology, Tianjin Institute of Urology, The Second Hospital of Tianjin Medical University, Tianjin, China; 5 Tianjin Human Sperm Bank, Tianjin Institute of Urology, The Second Hospital of Tianjin Medical University, Tianjin, China; 6 School of Pharmaceutical Science and Technology, Faculty of Medicine, Tianjin University, Tianjin, China; 7 Department of Urology, The First Affiliated Hospital of Jinzhou Medical University, Jinzhou, Liaoning China

**Keywords:** Renal cell carcinoma, Cancer metabolism

## Abstract

Renal cell carcinoma (RCC) is sometimes referred to as a “metabolic disease”, as nearly all types of RCC are associated with the reprogramming of glucose and lipid metabolism. Y-box binding protein 1 (YBX1) plays a crucial regulatory role in the development and progression of various cancers. In the early stages of our study, we analyzed the YBX1 binding proteins in 786-O cells using IP-MS and found that YBX1 is involved in the glycolysis process of RCC. Subsequent experiments showed that YBX1 is an oncogene that is significantly upregulated in RCC. Functionally, YBX1 promotes glycolysis in RCC, and both in vitro and in vivo experiments demonstrate that YBX1 contributes to the malignant progression of RCC. The correlation between YBX1 and Lactate Dehydrogenase A (LDHA) expression was predicted by bioinformatics and further explored in clinical RCC tissues. Mechanistically, YBX1 interacts with LDHA and co-localizes in the cytoplasm. CUT&Tag and functional experiments further revealed that YBX1 regulates LDHA through transcription. Additionally, YBX1 and LDHA activate the nuclear factor kappa-B (NF-κB) signaling pathway. Silencing the LDHA gene or using an LDHA inhibitor rescued the YBX1-mediated activation of the NF-κB signaling pathway and inhibited lactic acid production and RCC cell proliferation. In conclusion, these findings provide new insights into the oncogenic role of YBX1 in glycolysis and suggest that the YBX1-LDHA-NF-κB axis may represent a promising therapeutic target of RCC.

## Introduction

Renal cell carcinoma (RCC) is a malignant tumor originating from renal epithelium and is one of the most common tumors in the urinary system [1]. RCC accounts for more than 90% of renal malignancies, ranking second only to prostate cancer and bladder cancer among male urinary system malignant tumors [2]. Renal clear cell carcinoma (ccRCC), papillary renal cell carcinoma, and chromophobe RCC are the three most prevalent and deadly subtypes of RCC, accounting for 70–75% of all RCC cases [3]. VHL mutation is common in RCC, which usually leads to metabolic abnormalities [4, 5]. Unlike normal cells, most cancer cells generate energy through aerobic glycolysis, a phenomenon known as the “Warburg effect”, which is a key hallmark of cancer [6]. Therefore, it is urgent to explore new therapeutic targets to better understand the role of RCC metabolism and progression.

YBX1 is a multifunctional member of the cold shock domain protein family and is an important transcription factor. YBX1 exerts its transcriptional regulatory effects by binding to Y-box sequences located in the enhancer and promoter regions of numerous genes [7], thereby controlling the expression of genes implicated in cancer onset, progression, metastasis, and resistance to therapy. YBX1 is highly expressed in various cancers, including breast cancer [8], liver cancer [9], pancreatic cancer [10], and ovarian cancer [11]. It is significantly associated with poor prognostic outcomes across these diseases. Thus, YBX1 has emerged as a potential therapeutic target for various types of cancer. Our previous work has demonstrated that YBX1 is highly expressed in RCC and affects its progression [12, 13]. Recent reports have shown that YBX1 is associated with metabolic reprogramming and promotes tumor growth by enhancing glycolysis in bladder cancer [14]. In triple-negative breast cancer, YBX1 is also closely related to glycolytic genes [15]. Therefore, it is important to elucidate the potential mechanisms by which YBX1 regulates metabolic reprogramming in RCC.

LDHA is a crucial enzyme in aerobic glycolysis that catalyzes the conversion of pyruvate into lactate [16]. The expression of LDHA is increased in a variety of tumors [17] and plays a crucial role in regulating cellular metabolism and maintaining redox balance [18]. It has been demonstrated that LDHA is highly expressed in ccRCC and is associated with its poor prognosis [19]. C-Myc and hypoxia-inducible factor (HIF) have been shown to promote the transcription of LDHA [20]. However, the role of YBX1 in regulating LDHA and the mechanisms underlying this regulation remain poorly understood.

In RCC, the NF-κB pathway regulates cell proliferation, apoptosis and immune response [21]. In this study, we determined the specific molecular mechanisms by which YBX1 regulates glycolysis, proliferation, and metastasis in RCC. Our results demonstrate that the expression of YBX1 and LDHA in RCC is significantly associated with poor clinical outcomes. We found that LDHA interacts with YBX1, and LDHA is regulated by YBX1 transcriptional level. Additionally, YBX1 can activate the NF-κB pathway through LDHA to enhance glycolysis and proliferation in RCC.

## Methods

### Clinical samples and online data collection

We collected ccRCC tissues and adjacent normal tissue paraffin blocks from patients admitted to Tianjin Medical University Second Hospital between 2015 and 2019. Patient pathological data, including T stage, age, gender, tumor size, and Fuhrman grade, were gathered. All patients had total or partial surgical resection and received no preoperative therapies. After slicing the collected tissue paraffin blocks, immunohistochemical staining was performed to detect YBX1 and LDHA, and clinical data were analyzed to determine the correlation between YBX1 and LDHA with patient clinicopathological parameters. The evaluation of specimens was conducted by two pathologists through a double-blind method. This study was approved by the Institutional Review Board of Tianjin Medical University and was conducted in accordance with the principles outlined in the Declaration of Helsinki. Informed consent was obtained from each patient prior to their inclusion in this study.

In this study, we used UALCAN (http://ualcan.path.uab.edu/index.html) and the CPTAC analysis module of the online analysis database to obtain the expression levels of YBX1 and LDHA proteins in tumors [22]. This paper analyzed the correlation of *YBX1* and *LDHA* mRNA in KIRC using the Cancer Genome Atlas (TCGA) online database and examined the relationship between YBX1 and patient survival rates in KIRC [23]. GeneMANIA (http://genemania.org/) was employed to explore the co-expression of YBX1 and LDHA [24]. STRING (https://string-db.org) was used to analyze the co-expression of proteins YBX1 and LDHA [25]. The protein models from the AlphaFold protein structure database (https://alphafold.ebi.ac.uk/) [26] and the Protein Data Bank (PDB) (https://www.rcsb.org/pdb/) [27] were used for molecular docking analysis. YBX1 was sourced from the AlphaFold identifier AF-P67809-F1 and PDB identifier 5YTX, while LDHA was sourced from the AlphaFold identifier AF-P00338-F1. The ZDOCK server (https://zdock.wenglab.org/) was used to predict the most likely three-dimensional conformation when YBX1 and LDHA proteins bind, utilizing a fast Fourier transform (FFT) method [28]. The HDOCK server (http://HDOCK.phys.hust.edu.cn/) was used to make docking predictions between the YBX1 protein and the *LDHA* promoter DNA [29]. All structural diagrams were generated using PyMol.

### Cell lines and cultivation methods

All cell lines used in this study were purchased from the American Type Culture Collection (ATCC) and are mycoplasma-free, including human embryonic kidney cells HEK-293T, and human renal cancer cell lines ACHN and 786-O. Authenticate with short tandem repeat (STR) profiling. ACHN cells were cultured in MEM medium (Viva Cell, Germany), and 786-O and HEK-293T cells were cultured in DMEM medium (Viva Cell, Germany). All culture media were supplemented with 10% FBS (Viva Cell, Germany) and 1% PS (Viva Cell, Germany). Cells were cultured in a humidified incubator at 37 °C with 5% CO_2_.

### Stable cell line construction and transfection

HEK-293T cells were transfected using Lipofectamine 2000 (Invitrogen, USA) with lentiviral vectors, including pLKO.1-Scr (control), pLKO.1-shYBX1 (YBX1 knockdown), pCDH (control), pCDH-YBX1 (YBX1 overexpression), and pCDH-LDHA (LDHA overexpression), as well as lentiviral packaging plasmids (psPAX2 and pMD2.G). After 12–18 h of transfection, the medium was replaced with fresh medium containing 10% FBS. Lentivirus particles were collected, centrifuged, and filtered through a 0.45 μm filter 48 h post-transfection. Subsequently, 786-O and ACHN cells were infected with an appropriate volume of virus solution for 12–24 h. After 48 h, cells were selected with 2 μg/mL puromycin (Sangon, China) for 10–14 d. Cells were then maintained with 1 μg/mL puromycin to ensure survival. The expression of molecules in the stable cell lines was validated by western blot and qRT-PCR.

To investigate the interaction sites between YBX1 and LDHA, a series of YBX1 mutants, including the N-terminal domain and cold shock domain (1-129 amino acids), as well as portions of the CTD (130–205 aa or 206–324 aa) were used [13]. The GFP-YBX1 construct was a gift from the Genomics and Precision Medicine Key Laboratory (Chinese Academy of Sciences, Beijing, China). The Flag-tagged LDHA deletion mutants (Mailgene, China) include 1–163, 21–333 aa, deletion of 21–163 aa, and 21–163 aa [30]. Each experimental group was seeded with 786-O cells at a density of 60–70% on a 10 cm plate, and expression vectors were transiently transfected using Lipofectamine 2000 (Invitrogen, USA). After 6–8 h of transfection, the medium was replaced with DMEM containing 10% FBS. Structural domain immunoprecipitation experiments were performed 48 h post-transfection.

In rescue experiments, 6 μL of siRNA (20 μM) (Sangon, China) was transfected into 786-O cells using Lipofectamine 2000 (Invitrogen, USA). The sequences of siRNA are provided in Supplementary Table 1 [31, 32]. After 48 h of transfection, total RNA and protein were extracted, and LDHA knockdown was validated by western blot and qRT-PCR. LDHA inhibitor Oxamate (MedChemExpress, USA) (0–80 mmol/L, 48 h) was used to treat 786-O cells [33], and LDHA expression was validated by western blot.

### Western blot

Cell and tissue protein extracts were prepared using SDS protein lysis buffer and 1× cocktail (Roche, USA) at a 10:1 ratio. Protein concentration was determined using the BCA protein assay kit (Solarbio, China), with a bovine serum albumin (BSA) standard curve for quantification. SDS-PAGE gels (8–12% separation gel and stacking gel) were prepared according to the molecular weight of the proteins, and electrophoresis was performed at 80 V. Once the samples had run out of the separation gel and the marker (Thermo, USA) bands had been separated, the voltage was increased to 120 V. The gel was transferred to a PVDF membrane (Millipore, USA) at 250 mA for 2 h. The membrane was blocked with 5% non-fat dry milk (BD, USA) at room temperature for 2 h. Primary antibodies were incubated overnight at 4 °C. The appropriate secondary antibodies were incubated at room temperature for 1 h. ECL chemiluminescent substrate (Millipore, Germany) was used for exposure under a chemiluminescence imaging system. The details of antibodies used are provided in Supplementary Table 2.

### Real-time fluorescence quantitative PCR (qRT-PCR)

Total RNA was extracted using Trizol reagent (Invitrogen, USA) and DNA extraction buffer (Acmec, China), cDNA was synthesized using HiScript Q RT SuperMix (Vazyme, China) according to the manufacturer’s instructions. The cDNA, primers (Sangon, China), and 2× ChamQ Universal SYBR qPCR Master Mix (Vazyme, China) were prepared in the appropriate proportions for qRT-PCR. The expression of target genes was normalized to GAPDH and analyzed using the 2^−ΔΔCt^ method. The primers for qRT-PCR are provided in Supplementary Table 3.

### Lactate production assay

Lactate levels were measured using a lactate assay kit (Nanjing Jiancheng Bio, China) according to the manufacturer’s instructions. Cells were collected, washed twice with PBS, and then centrifuged at 1000 rpm for 5 min to collect the pellet. The pellet was resuspended in PBS, and the cells were disrupted by sonication. The supernatant was obtained by centrifugation at 4000 rpm for 10 min. Before use, the enzyme stock solution and enzyme buffer were mixed in a 1:100 volume ratio to prepare the enzyme working solution. For each well, 0.02 mL of sample/distilled water/standard, 1 mL of enzyme working solution, and 0.2 mL of color reagent were added. The mixture was incubated at 37 °C for 10 min. After adding 2 mL of termination solution, the mixture was thoroughly mixed, and the absorbance at 530 nm was measured using a microplate reader (Biotek, USA). The lactate content was calculated based on the protein concentration.

### ATP level assay

ATP levels were measured using an ATP assay kit (Biotium, China) following the manufacturer’s instructions. For each well of a 6-well plate, 200 μL of lysis buffer was added, and the plate was shaken to ensure complete contact and cell lysis. After lysis, the sample was centrifuged at 12,000 g for 5 min at 4 °C to collect the supernatant. An ATP standard solution was diluted in ATP assay lysis buffer to generate a concentration gradient of 0, 0.1, 0.5, 1, 3, 6, and 10 μM. The ATP assay reagent dilution buffer was mixed in a 1:9 ratio to prepare the ATP working solution. Each well received 100 μL of the ATP working solution, which was then incubated at room temperature for 3–5 min. Then, 20 μL of sample or standard was added to each well, mixed quickly, and after at least a 2 s interval, the relative light units (RLU) were measured using a luminometer (Promega, USA). ATP concentration in the sample was calculated based on the standard curve. Protein concentration was determined using the BCA method (Solarbio, China), and ATP levels were expressed as nmol/mg protein.

### Extracellular acidification rate (ECAR) and oxygen consumption rate (OCR) measurement

According to the user manual, extracellular acidification rate (ECAR) and oxygen consumption rate (OCR) were measured using the Seahorse XFe24 extracellular flux analyzer (Agilent Technologies, USA). The cells were counted at 1–8 × 10^4^ cells per well in a volume of 100 µL and plated onto an XFe24 culture plate. After attachment, 150 µL of culture medium without antibiotics was added, and the plates were incubated overnight. Each well was subsequently filled with 1 mL of Seahorse XF Calibrant solution and placed in a 37 °C incubator without CO_2_ overnight for probe hydration. Prior to testing, XF Assay Buffer was prepped, adjusted to a pH of 7.35–7.40 at 37 °C, filtered through a 0.22 µm filter, and aliquoted into 50 mL centrifuge tubes for storage at 4 °C. For the ECAR assay, drugs required included Glucose (Sigma, Germany), 2-Deoxy-D-glucose (2-DG) (Sigma, Germany), and Oligomycin (MedChemExpress, USA), each dissolved in 2 mL of specialized XF Assay Buffer for glycolytic stress assay and vortex mixed. For the OCR assay, Oligomycin (MedChemExpress, USA) and FCCP (Absin, China) were separately dissolved in 2 mL of mitochondrial stress assay-specific XF Assay Buffer, while Rotenone (Sigma, Germany) and Antimycin-A (Biovision, USA) were combined in 2 mL of XF Assay Buffer and vortex mixed for later use. The cells were washed three times with XF Assay Buffer, followed by the addition of 500 µL of XF Assay Buffer. The cells were then incubated in a 37 °C incubator without CO_2_ for 1 h. Following this incubation, the respective pre-prepared drugs were added to the wells, and calibration was performed on the instrument. The hydration plate was removed from the probe assembly, and the probe plate was inserted into the cell plate for measurement. After testing, cells were lysed, and protein concentration was determined using the BCA method for normalization purposes.

### LDH activity assay

LDH activity was measured using an LDH assay kit (Nanjing Jiancheng Bio, China) following the manufacturer’s instructions. Cells were collected, ensuring at least 1 × 10^6^ cells per sample, and resuspended in 0.3 mL of PBS. Cells were sonicated in an ice bath, followed by centrifugation at 4000 rpm for 10 min to obtain the supernatant. For the assay, 16 μL of sample was added to the measurement wells, 4 μL of distilled water and 16 μL of standard solution to the standard wells, 4 μL of distilled water and 16 μL of sample to the control wells, and 20 μL of distilled water to the blank wells. Then, 20 μL of substrate buffer was added to each well, followed by 4 μL of coenzyme I in the measurement wells. The mixture was incubated at 37 °C for 15 min. Afterward, 20 μL of 2,4-dinitrophenylhydrazine was added to each well, and the mixture was incubated at 37 °C for another 15 min. Finally, 200 μL of 0.2 mol/L NaOH solution was added, the mixture was mixed thoroughly, and it was allowed to sit at room temperature for 5 min. Absorbance at 440 nm was measured using a microplate reader (Biotek, USA), and LDH activity was calculated accordingly.

### Cell proliferation assay

In the colony formation assay, cells were seeded at a density of 500 cells per well in a 6-well plate. The medium was changed every three days, and the cells were cultured for 10–14 d. After washing with PBS, the cells were fixed with 4% paraformaldehyde and stained with crystal violet. Following imaging, the colony area was measured using Image J software. In the CCK-8 proliferation assay, a Cell Counting Kit-8 (GeneRun, China) was utilized. Cells were seeded at a density of 1000 cells per well in a 96-well plate. After incubation for 0, 24, 48, 72, and 96 h, 10 μL of CCK-8 reagent was added to each well. The plate was then incubated at 37 °C in the dark for 2 h, and absorbance at 450 nm was measured using a microplate reader.

### Cell migration and invasion assay

In the Transwell migration assay, 600 μL of basic medium containing 20% FBS was added to the lower chamber of the Transwell plate (LABSELECT, China). A cell suspension of 200 μL containing ACHN or 786-O cells, cultured in either DMEM or MEM basic medium, was added to the upper chamber. ACHN cells were seeded at a density of 1.5 × 10^5^ cells per chamber, while 786-O cells were seeded at a density of 6 × 10^4^ cells per chamber. In the Transwell invasion assay, 50 μL of Matrigel (1:10; BD, USA) was uniformly spread inside the Transwell chamber prior to adding cells. The plate was incubated at 37 °C for 2 h. The subsequent steps were identical to those in the migration assay. After allowing time for cells to migrate to the lower chamber, the wells were washed with PBS, fixed with 4% paraformaldehyde, and stained with crystal violet. After washing with PBS, non-migrated cells on the upper side of the membrane were carefully wiped off. Five random fields at 200× magnification were selected to count the number of cells.

### Xenograft transplantation

Animal experiments were conducted following the protocol approved by the Ethics Committee of Tianjin Medical University (TMUaMEC 2025010). A total of 20 BALB/c nude mice (6–8 weeks old) were randomly divided into two groups (equal numbers of males and females). All animals are kept in a maximum of four per cage and are provided with food and water ad libitum and are kept in a temperature-controlled environment with a 12/12 h light and dark cycle, with light starting at 07:00. The 786-O-SCR or 786-O-shYBX1 cells, stably expressing luciferase, were counted and mixed with Matrigel (BD, USA) at a 1:1 ratio. The mixture containing 2 × 10^6^ cells was injected into the left kidney of anesthetized nude mice, and the incision was sutured. After 8–10 weeks of feeding, luciferin substrate (Promega, USA) was injected intraperitoneally into the nude mice. Ten min later, the mice were anesthetized, and the bioluminescence intensity was detected using an IVIS imaging system (Perkin Elmer, USA) to assess the tumor size. The mice were then sacrificed, the kidney tissue was removed, and the left kidney was subtracted from the weight of the matching right kidney to obtain the tumor weight. The tissue was then fixed in 4% formaldehyde, embedded in paraffin, cut into continuous 5.0 μm sections, and immunohistochemically stained.

### Immunohistochemistry (IHC)

This study collected cancerous tissues and adjacent tissues from 63 patients diagnosed with RCC at the Second Hospital of Tianjin Medical University. Data, including hospitalization number, gender, age, tumor size, TNM staging, and Fuhrman grading, were collected. Tissue sections were cut to a thickness of 2 μm, deparaffinized, and rehydrated. Antigen retrieval was performed by heating in 0.01 M sodium citrate buffer (pH 6.0) (Solarbio, China) at 98 °C for 20 min. The sections were then treated with 3% hydrogen peroxide for 15 min, followed by blocking with 5% BSA solution for 1 h. The sections were incubated overnight at 4 °C with primary antibodies for YBX1 (Abcam, UK) and LDHA (Abcam, UK). After warming to room temperature, the sections were incubated with secondary antibody (ZSGB-BIO, China) for 1 h at room temperature. HRP-DAB reagent (ZSGB-BIO, China) was used for color development, and the sections were counterstained with hematoxylin, dehydrated, and mounted. The staining was scored based on the percentage of tumor cells exhibiting positive staining (staining area) and the intensity of staining. The mean optical density was employed to calculate the relative expression of YBX1, LDHA, Ki-67, p65 and p-p65 using Image J software. The details of antibodies used are provided in Supplementary Table 2.

### Immunofluorescence (IF)

Cells were seeded in a 12-well plate containing cover slips and cultured overnight. After washing with PBS, cells were fixed with precooled methanol at 4 °C for 10 min, followed by permeabilization with 0.2% Triton X-100 (Solarbio, China) prepared in PBS at room temperature for 10 min. The cells were then blocked with 3% BSA in PBS at room temperature for 1 h. Antibodies YBX1 (Mouse; Santa Cruz Biotechnology, USA) and LDHA (Rabbit; Abcam, UK) diluted in 3% BSA were added, and the cells were incubated overnight at 4 °C. Next, Alexa-Fluor 488 AffiniPure Goat Anti-Rabbit IgG (Abbkine, China) and Alexa-Fluor 594 AffiniPure Goat Anti-Mouse IgG (Abbkine, China) were added and incubated in the dark at room temperature for 1 h. DAPI was used to stain the nuclei at room temperature in the dark for 10 min. Finally, the cover slips were mounted on slides using an anti-fade mounting medium (Solarbio, China), and subcellular localization of YBX1 protein (red), LDHA protein (green), and nuclei (blue) was detected using a fluorescence confocal microscope (Zeiss Lsm800, Germany). The details of antibodies used are provided in Supplementary Table 2.

### Co-immunoprecipitation (Co-IP)

Cells grown in 10 cm culture dishes were collected from each group and lysed in IP buffer (40 mM Tris-Cl, 120 mM NaCl, 1% Triton X-100) supplemented with phosphatase inhibitors (1 mM NaF, 1 mM Na_3_VO_4_) and a cocktail (Roche, USA). The lysates were rotated at 4 °C for 30 min and centrifuged at 12,000 rpm for 30 min at 4 °C to obtain clarified lysates. To Dynabeads Protein A magnetic beads (Invitrogen, USA), 4 μg of negative control IgG antibody (Rabbit; Cell Signaling Technology, USA) and target antibodies YBX1 (Rabbit; Abcam, UK), LDHA (Rabbit; Abcam, UK), and GFP (Rabbit; SIGMA, Germany) were added and mixed thoroughly, followed by slow rotation at 4 °C for 6 h. The total protein concentration of the lysates was measured using the BCA protein assay (Solarbio, China), and an appropriate amount of protein was reserved as Input. The remaining supernatant was equally distributed into tubes containing each antibody, gently mixed by pipetting, and further incubated overnight at 4 °C. Cell lysates transfected with LDHA domain plasmids were immunoprecipitated using anti-DYKDDDDK magnetic agarose (Thermo Scientific, USA). After washing the magnetic beads five times with IP buffer, the immunoprecipitates were resuspended in 2× loading buffer, boiled, and analyzed by western blot. The details of antibodies used are provided in Supplementary Table 2.

### Cleavage under targets and tagmentation (CUT&Tag) assay

The CUT&Tag detection was performed using the Hyperactive Universal CUT&Tag Assay Kit for Illumina Pro (Vazyme, China). ACHN cells (2 × 10^5^ per group) were washed with 500 μL Wash Buffer. After treatment with TF Enhancer, each sample was resuspended in 100 μL Wash Buffer. A portion of the suspension (100 μL) was transferred to an 8-tube strip containing activated ConA Beads Pro, inverted and mixed thoroughly, and incubated at room temperature for 10 min, with gentle inversion 2–3 times during incubation. After removing the supernatant, 50 μL of Antibody Buffer was added to resuspend the beads-bound cells, followed by the addition of 1 μg of YBX1 antibody (Proteintech, USA) and Rabbit IgG (Cell Signaling Technology, USA), and left to stand overnight at 4 °C. The next day, careful disposal of the primary antibodies was performed, and 0.5 μL of Mouse Anti-Rabbit IgG (Vazyme, China) was added to the cells, diluted with 50 μL Dig-wash Buffer. The cells were then gently rotated and incubated for 1 h at room temperature, followed by three washes with 200 μL Dig-wash Buffer. Subsequently, 2 μL of pA/G-Tnp Pro and 98 μL of Dig-300 Buffer were added to each sample, and after 1 h of rotation at room temperature, the samples were washed three times with 200 μL Dig-300 Buffer. Next, 10 μL of 5× TTBL was mixed with 40 μL of Dig-300 Buffer, and the mixture was incubated at 37 °C in a PCR machine for 1 h. After adding 2 μL of 10% SDS and mixing gently, the samples were incubated at 55 °C for 10 min followed by 80 °C for 2 h. Following this, 50 μL of DNA Extract Beads Pro were added, mixed thoroughly, and incubated at room temperature for 20 min. The supernatant was discarded, and the beads were washed three times with 200 μL of 1× B&W Buffer before being resuspended in 15 μL of ddH_2_O. For library amplification, 15 μL of purified DNA was mixed with 25 μL of 2× CAM and 5 μL of i5 and i7 index primers from the TruePrep Index Kit V2 for Illumina (Vazyme, China) and subjected to PCR reactions. To purify the PCR products, 100 μL of VAHTS DNA Clean Beads (Vazyme, China) were added and incubated at room temperature for 5 min. The magnetic beads were washed twice with 200 μL of fresh 80% ethanol, and 22 μL of ddH_2_O was added for elution. All CUT&Tag libraries were sequenced using the Illumina NovaSeq 6000 platform in PE150 mode by Novogene (China). The CUT&Tag reads were aligned to the human genome (UCSC hg38) using Bowtie2.

### Chromatin immunoprecipitation (ChIP-qPCR)

Three pairs of primers (Sangon, China) for YBX1 binding sites in the *LDHA* promoter region were designed using the JASPAR database. RCC cells were processed using the ChIP Assay Kit (Beyotime, China). The cells were fixed with 1% formaldehyde for 10 min, followed by the addition of 1.25 mol/L glycine to terminate the crosslinking process. Cells were collected and sonicated to break the chromatin, followed by centrifugation at 4 °C, 4000 rpm for 10 min. 4 μg of YBX1 antibody (Proteintech, USA) and Rabbit IgG (Cell Signaling Technology, USA) were added to bind to the target protein-DNA complex, and incubation was carried out overnight at 4 °C. The next day, 25 μL of Dynabeads Protein A magnetic beads (Invitrogen, USA) were added to capture the antibody-target protein-DNA complex, followed by washing to remove non-specific bindings. The complex was then eluted to obtain the enriched target protein-DNA complex, which was subjected to crosslink reversal at 65 °C overnight. DNA fragments were purified using a DNA recovery kit (Vazyme, China). ChIP-qPCR was performed using the ChamQ Universal SYBR qPCR Master Mix (Vazyme, China). The primers for ChIP-qPCR are provided in Supplementary Table 4.

### Dual-luciferase reporter assay

RCC cells were seeded into 48-well plates and cultured to 60-70% confluence. Subsequently, 200 ng of pGL3-luc luciferase plasmid driven by the *LDHA* promoter (Integrated Biotech Solutions, China) or relevant pathway luciferase plasmids, along with 50 ng of pRL-TK Renilla luciferase plasmid (Promega, USA), were co-transfected into the cells using Lipofectamine 2000 (Invitrogen, USA). After 48 h of transfection, cells were harvested and the luciferase activities were measured using the Dual-Luciferase Reporter Assay System (Promega, USA), following the manufacturer’s protocol. Luciferase activity was normalized to Renilla luciferase activity to account for the background signal.

### Statistical analysis

Statistical analysis was performed using GraphPad Prism 9.0. Unpaired or Paired two-sided Student’s *t*-test, Log-rank test, Spearman correlation analysis, One-way ANOVA, and Two-way ANOVA were applied according to the experimental sample type. Statistical significance was determined with the following criteria: *P < 0.05 was considered significant. ns, *P* ≥ 0.05, *P < 0.05, **P < 0.01, ***P < 0.001, ****P < 0.0001.

## Results

### High expression of YBX1 in ccRCC and its association with metabolism

**Fig. 1:**
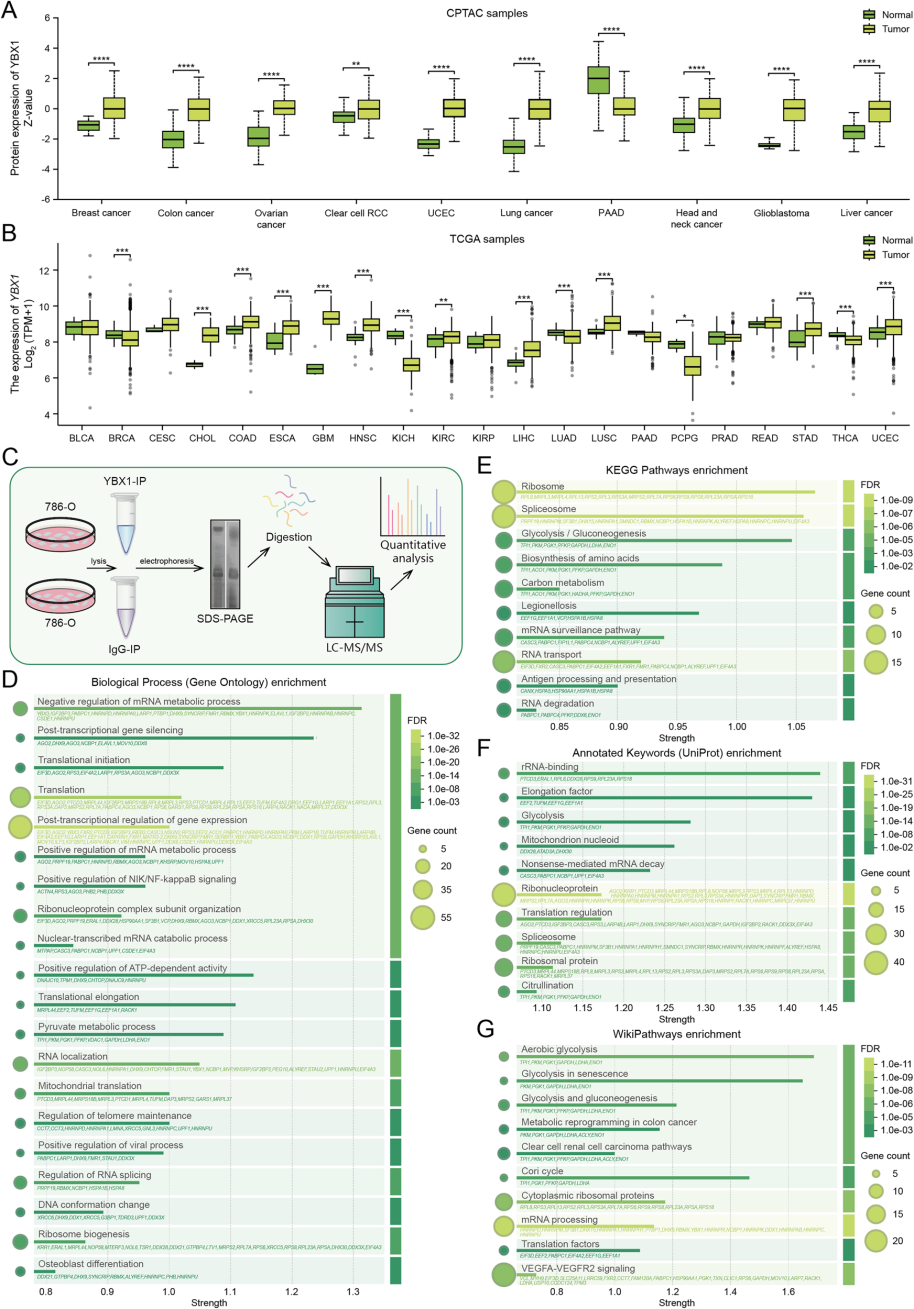
YBX1 interacting proteins related to metabolism in ccRCC. **A** Protein levels of YBX1 in 10 different types of tumors from the CPTAC database. **B** mRNA levels of *YBX1* in 21 different types of tumors from the TCGA database. **C–G** Enrichment functional analysis of proteins interacting with YBX1 in ccRCC cell line 786-O through Co-IP pull-down and identified by mass spectrometry. **C** Mass spectrometry identification schematic. **D** Gene Ontology Biological Process (GO-BP) enrichment. **E** KEGG pathway enrichment. **F** UniPort annotation keywords. **G** Wiki Pathways enrichment analysis. **P < 0.01, ***P < 0.001, ****P < 0.0001. Unpaired two-sided Student’s *t*-test in A and B. Data are presented as mean ± SD.

To investigate the role of YBX1 in ccRCC, we analyzed the expression of YBX1 across various cancers using the UALCAN database. The results indicated that, compared to matched normal tissues, YBX1 protein expression was elevated in multiple tumors, including ccRCC (Fig. 1A). Analysis of the TCGA tumor proteomics dataset revealed that *YBX1* mRNA expression was also increased in ccRCC relative to other tumor types (Fig. 1B). This finding is consistent with our previous study, which demonstrated that YBX1 protein levels are higher in ccRCC than in adjacent normal tissues [13]. To explore the biological functions of YBX1 in ccRCC, we identified endogenous interacting proteins of YBX1 in 786-O ccRCC cells through LC − MS/MS analysis [12] (Fig. 1C). GO-BP analysis showed that these interacting proteins were primarily involved in processes, such as transcriptional regulation, translation, positive regulation of NIK/NF-κB signaling, and ATPase activity (Fig. 1D). KEGG pathway enrichment results indicated that the interacting proteins clustered around pathways related to glycolysis/gluconeogenesis and ribosome function (Fig. 1E). UniProt keyword annotation highlighted glycolytic enzymes and ribosomal proteins (Fig. 1F), while WikiPathways similarly highlighted enrichment in aerobic glycolysis, glycolysis during the aging process, gluconeogenesis, and pathways associated with ccRCC (Fig. 1G). Previous reports have shown that YBX1 acts as a key molecule regulating glycolysis-related genes in triple-negative breast cancer [15] and promotes glycolysis in bladder cancer [14]. However, the relationship between YBX1 and energy metabolism in ccRCC remains to be elucidated. Glycolysis plays a critical role in the onset, progression, and therapeutic response of ccRCC, suggesting that YBX1 may significantly influence ccRCC metabolic processes via glycolysis-related proteins (e.g., LDHA, PKM, ENO1).

### YBX1 involvement in glycolytic metabolism in ccRCC

**Fig. 2:**
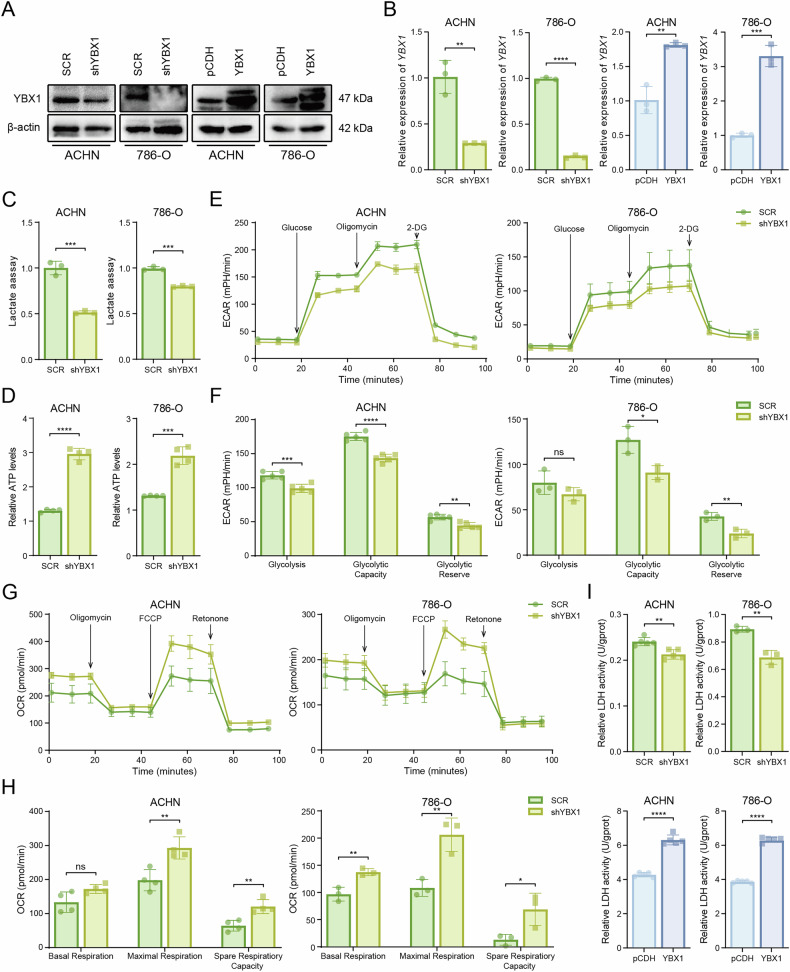
YBX1 regulates glycolytic metabolism in ccRCC. **A** Western blot validation of YBX1 protein expression upon knockdown and overexpression efficiency in ACHN and 786-O cells. **B** qRT-PCR validation of *YBX1* mRNA expression upon knockdown and overexpression efficiency in ACHN and 786-O cells. **C** Lactate production upon YBX1 knockdown in ACHN and 786-O cells. **D** ATP production detection upon YBX1 knockdown in ACHN and 786-O cells. **E** Real-time monitoring of extracellular acidification rate (ECAR) in ACHN and 786-O cells after YBX1 knockdown, measured using a Seahorse Bioscience Analyzer. **F** Quantitative analysis of glycolytic capacity and glycolytic reserve. **G** Real-time monitoring of oxygen consumption rate (OCR) in ACHN and 786-O cells after YBX1 knockdown, measured using a Seahorse Bioscience Analyzer. **H** Quantitative analysis of mitochondrial respiratory capacity and respiratory reserve. **I** Lactate dehydrogenase (LDH) activity detection upon YBX1 knockdown and overexpression in ACHN and 786-O cells. *P < 0.05, **P < 0.01, ***P < 0.001, ****P < 0.0001. Unpaired two-sided Student’s *t*-test in **B**–**D**, **F**, **H** and **I**. Data are presented as mean ± SD.

To further investigate the specific pathways through which YBX1 influences energy metabolism in ccRCC, we constructed stable YBX1 knockdown and overexpression ccRCC cell lines (ACHN and 786-O) using lentiviral infection. The efficiency was confirmed by western blot and qRT-PCR (Fig. 2A, B). First, we measured the production of lactate and ATP. Compared with the control group, YBX1 knockdown cells exhibited reduced lactate production but increased ATP levels (Fig. 2C, D). Using a glycolytic stress assay, we evaluated glycolytic capability by measuring the ECAR. The results demonstrated significant decrease in glycolytic activity in YBX1-knockdown cells (Fig. 2E). Treatment with oligomycin, an H^+^-ATP synthase inhibitor, blocks oxidative phosphorylation and electron transport chain activity. Upon oligomycin treatment, the increased acid production reflected glycolytic potential, while the total value represented maximal glycolytic capacity. Both maximal glycolytic capacity and glycolytic reserve were reduced in YBX1-knockdown cells (Fig. 2F). Using a mitochondrial stress assay, we assessed mitochondrial respiratory capacity via OCR measurements. YBX1 knockdown enhanced oxidative phosphorylation in ccRCC cells (Fig. 2G). When treated with FCCP, a respiratory chain uncoupler, the increased oxygen consumption indicated maximal respiratory capacity. The respiratory potential was calculated as the relative increase from baseline. YBX1-knockdown cells showed significantly greater maximal respiratory capacity and respiratory potential than controls (Fig. 2H), suggesting that YBX1 may suppress oxidative phosphorylation. Since lactate dehydrogenase (LDH) plays key roles in glycolysis by catalyzing lactate-pyruvate interconversion, and its activity correlates with tumor progression, we measured LDH activity. YBX1 knockdown decreased LDH activity, whereas overexpression increased it (Fig. 2I), indicating that YBX1 regulates ccRCC glycolysis via LDHA modulation.

### YBX1 promotes ccRCC progression

**Fig. 3:**
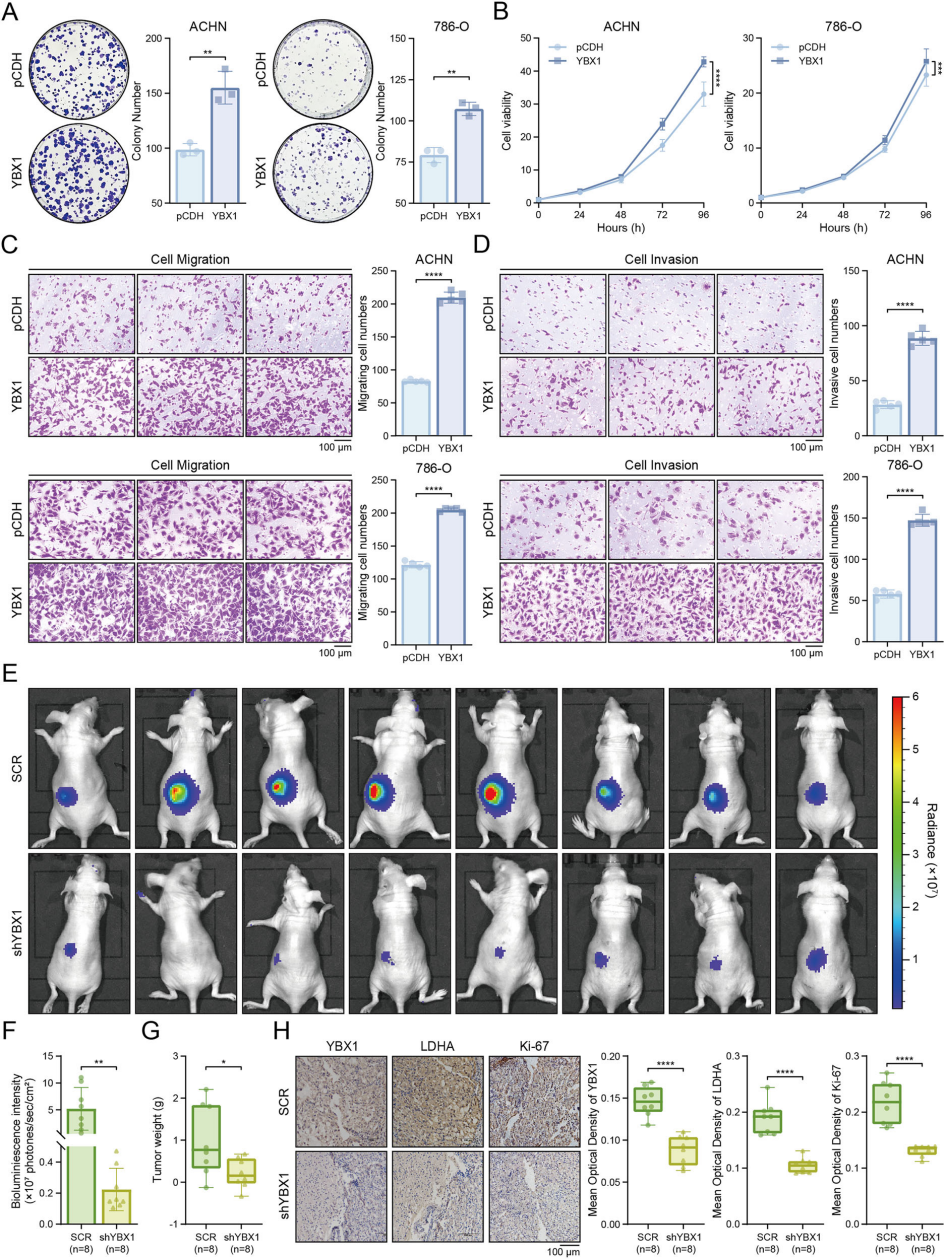
YBX1 promotes progression of ccRCC. **A**, **B** Effects of YBX1 overexpression on the proliferation of ACHN and 786-O cells. **A** Colony formation assay. **B** CCK-8 cell proliferation assay. **C, D** Effects of YBX1 overexpression on the migration and invasion of ACHN and 786-O cells. **C** Transwell cell migration assay. **D** Transwell cell invasion assay. **E** Detection of bioluminescence intensity of 8 pairs of tumors in nude mice using an in vivo imaging system. **F** Quantification analysis of bioluminescence intensity at the site of renal orthotopic tumors. **G** Statistical analysis of in situ tumor weight, expressed as the weight of the left minus the corresponding right kidney of nude mice. **H** Immunohistochemical staining and quantification to detect protein expression of YBX1, LDHA and Ki-67 in tumor tissues from control and YBX1 knockdown groups in nude mice. *P < 0.05, **P < 0.01, ***P < 0.001, ****P < 0.0001. Unpaired two-sided Student’s *t*-test in **A**, **C**, **D**, **F**, **G** and **H**. Two-way ANOVA with correction for multiple comparisons in B. Data are presented as mean ± SD.

To investigate YBX1’s role in renal cancer progression, we assessed its effects on cell proliferation, migration, and invasion. The colony formation assay demonstrated that YBX1 overexpression significantly increased ccRCC cell clonal growth compared with controls (Fig. 3A). CCK-8 assays confirmed enhanced proliferation in YBX1-overexpressing cells (Fig. 3B), supporting YBX1’s critical role in ccRCC growth. Since tumor progression involves both proliferation and metastatic potential, we evaluated migration and invasion capabilities. Transwell assays showed significantly more migrating (Fig. 3C) and matrigel-invading (Fig. 3D) cells in YBX1-overexpressing groups than controls, indicating YBX1 regulates ccRCC metastasis. For in vivo validation, we established orthotopic xenografts by implanting luciferase-tagged 786-O cells (control or YBX1-knockdown) into nude mice kidney capsules. After 8–10 weeks, in vivo imaging revealed significantly lower tumor bioluminescence in YBX1-knockdown mice (Fig. 3E, F), and the weight of the tumors in situ was also smaller than that of the control group (Fig. 3G), suggesting suppressed tumor growth. To further explore the potential association between YBX1 and LDHA, we excised tumor tissues from the nude mice for histological processing, including tissue embedding and sectioning. Immunohistochemical analysis of YBX1, LDHA and Ki-67 expression revealed that YBX1 knockdown led to a significant reduction in LDHA and Ki-67 protein levels (Fig. 3H). These results demonstrate that YBX1 not only promotes renal cancer growth in vivo but also exhibits a positive correlation with LDHA expression, suggesting a functional relationship between these two proteins in ccRCC progression.

### High expression of YBX1 and LDHA proteins in ccRCC tissues and their positive correlation

**Fig. 4:**
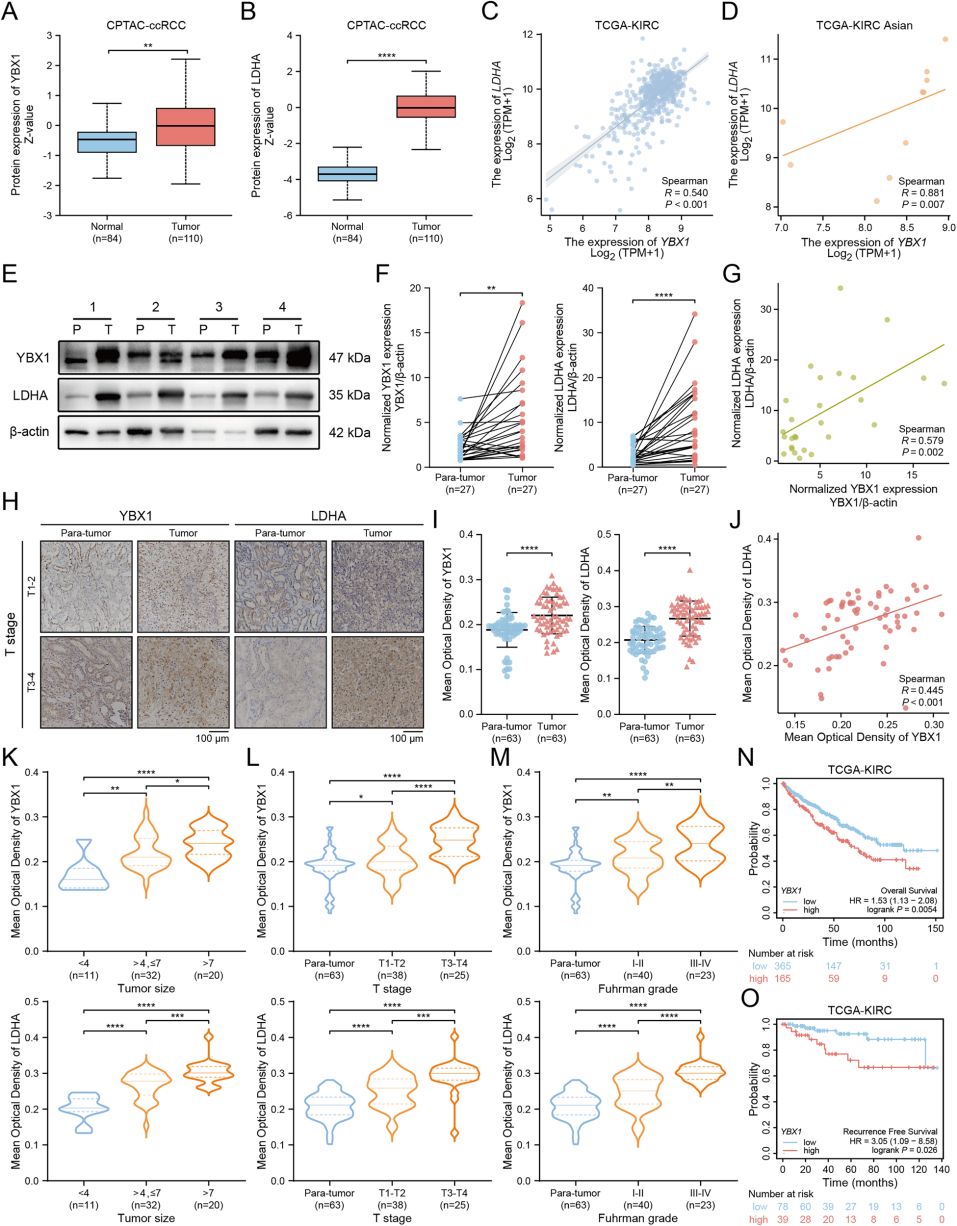
YBX1 and LDHA proteins are highly expressed and correlate positively in ccRCC tissues. **A, B** Protein expression levels of YBX1 (**A**) and LDHA (**B**) in ccRCC from the CPTAC database. **C, D** Correlation of gene expression between *YBX1* and *LDHA* in ccRCC from the TCGA database (**C**) and in an Asian population (**D**). **E** Western blot analysis of YBX1 and LDHA protein expression in tumor (T) and paired adjacent non-tumor (P) tissues from 27 ccRCC patients, showing representative images from 4 pairs of tissues. **F** Quantification of YBX1 and LDHA proteins in ccRCC and adjacent non-cancerous tissues from 27 cases using Image J software, normalized to β-actin. **G** Correlation of YBX1 and LDHA protein expression in tumor tissues from 27 ccRCC patients. **H-M** Immunohistochemical staining to assess the expression of YBX1 and LDHA in 63 pairs of ccRCC and adjacent tissues. **H, I** Representative immunohistochemical staining images and quantification of IHC staining using Image J software. **J** Correlation of quantified immunohistochemical staining of YBX1 and LDHA in tumor tissues of 63 ccRCC patients. **K-M** Levels of YBX1 and LDHA in ccRCC patients stratified by tumor size, T stage and Fuhrman grade. **N**, **O** Impact of *YBX1* expression on overall survival (OS) (**N**) and recurrence free survival (RFS) (**O**) in ccRCC patients from the TCGA database. **P < 0.01, ***P < 0.001, ****P < 0.0001. Unpaired two-sided Student’s *t*-test in **A**, **B**, **K**, **L** and **M**. Spearman correlation statistics in **C**, **D**, **G** and **J**. Paired *t*-test in F and I. Log-rank test in N and O. Data are presented as mean ± SD.

Analysis of the CPTAC database revealed significantly elevated levels of both YBX1 and LDHA proteins in ccRCC (Fig. 4A, B). TCGA data analysis demonstrated a positive correlation between *YBX1* and *LDHA* gene expression (Fig. 4C), with a particularly strong correlation in the Asian population (Fig. 4D). To validate these findings, we examined 27 paired clinical ccRCC samples, confirming significantly higher YBX1 and LDHA protein expression in tumor tissues versus adjacent normal tissues by western blot (Fig. 4E, F), with positive correlation between the two proteins (Fig. 4G). Immunohistochemical staining of 63 pairs of clinical ccRCC samples revealed that both YBX1 and LDHA were markedly upregulated in ccRCC tissues (Fig. 4H, I), and their expression showed a positive correlation (Fig. 4J). Clinical pathological information from the patients was collected, and statistical analysis was performed to assess the correlation between YBX1 and LDHA expression and clinical pathological features. The results showed that high expression of YBX1 and LDHA was associated with tumor size (Fig. 4K), T stage (Fig. 4L), and Fuhrman grade (Fig. 4M). Elevated YBX1 and LDHA levels were positively correlated with advanced stages of ccRCC. Additionally, we analyzed the relationship between YBX1 expression and survival in ccRCC patients using the TCGA database. The results showed that low expression of YBX1 was associated with better overall survival (OS) (Fig. 4N) and recurrence-free survival (RFS) (Fig. 4O) in ccRCC patients. In conclusion, these results indicate that YBX1 and LDHA are upregulated in ccRCC and that their expression exhibits a positive correlation. High expression of YBX1 and LDHA is associated with disease progression and poor prognosis in ccRCC.

### Interaction between YBX1 and LDHA

**Fig. 5:**
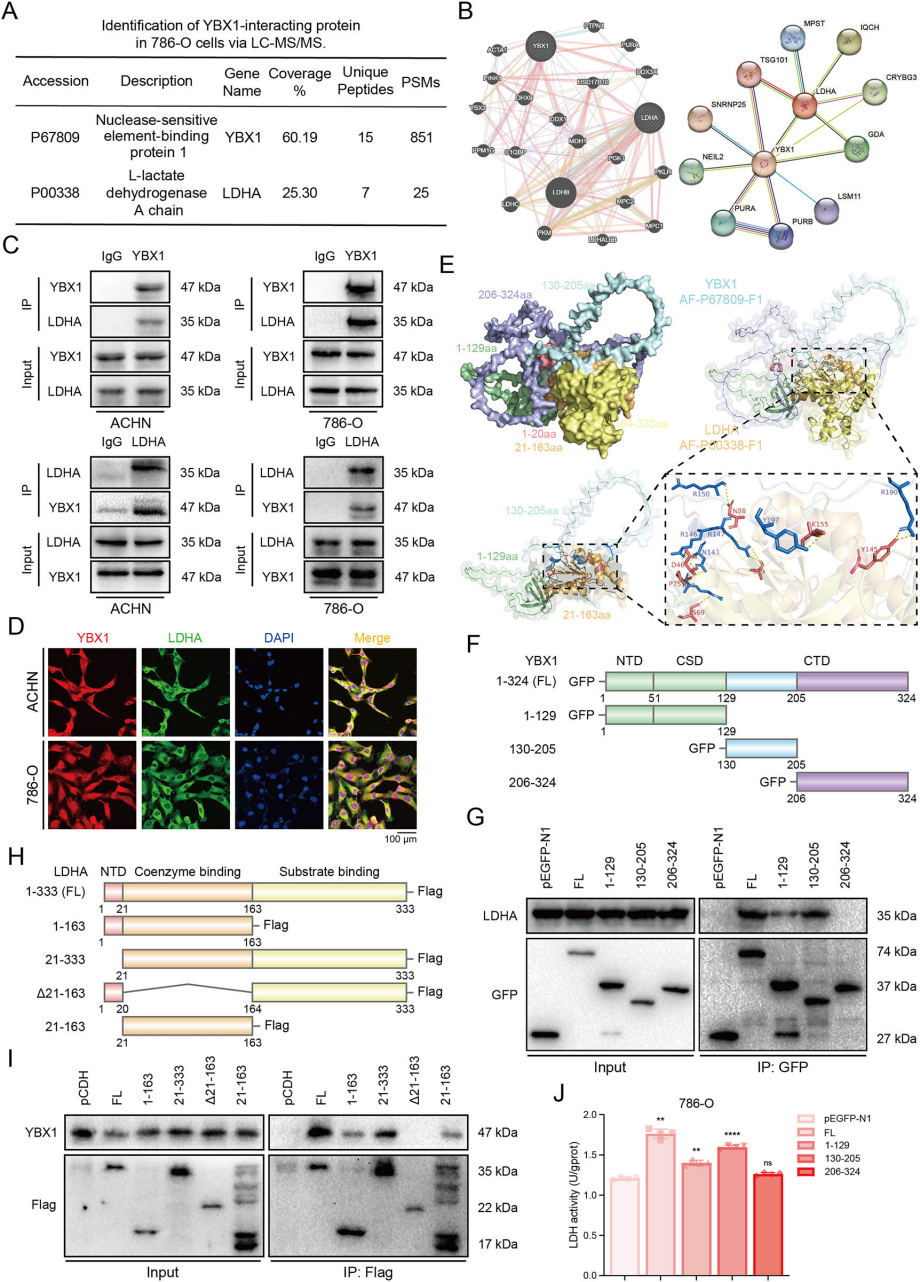
Interaction between YBX1 and LDHA. **A** Identification of LDHA as a potential YBX1-interacting protein by IP/MS analysis. **B** Protein-protein interaction (PPI) network diagram of YBX1 and LDHA from the GeneMANIA and STRING databases. **C** Co-IP assay to verify the YBX1-LDHA interaction in ccRCC cell lines. **D** Immunofluorescence staining to examine the expression of YBX1 and LDHA in ACHN and 786-O cell lines, with confocal microscopy used to observe the co-localization of YBX1 and LDHA. **E** Molecular docking analysis of the YBX1-LDHA interaction. **F** Schematic diagram of GFP-tagged YBX1 structural domain peptide fragments. **G** 786-O cells transfected with GFP-tagged YBX1 or its truncation mutants for 48 h, followed by Co-IP using anti-GFP antibody. **H** Schematic representation of Flag-tagged LDHA domain protein peptides. **I** 786-O cells transfected with Flag-tagged LDHA or its truncation mutants for 48 h, followed by Co-IP using anti-Flag antibody. **J** Assessment of the impact on LDH activity after transfecting YBX1 or its truncated mutants into 786-O cells for 48 h. **P < 0.01, ****P < 0.0001, ns: no significant difference. One-way ANOVA with correction for multiple comparisons in J. Data are presented as mean ± SD.

In mass spectrometry analysis of immunoprecipitated products from the ccRCC cell line 786-O, we identified the LDHA protein (Fig. 5A). Subsequently, we explored the co-expression of YBX1 and LDHA proteins through the GeneMANIA and STRING databases, which revealed that YBX1 and LDHA are co-expressed (Fig. 5B). To investigate the mechanism by which YBX1 regulates LDHA expression, we performed Co-IP experiments in ACHN and 786-O cells using YBX1 antibody-conjugated magnetic beads to pull down LDHA. Reciprocal Co-IP with an LDHA antibody confirmed that YBX1 interacts with LDHA in ccRCC cells (Fig. 5C). Additionally, immunofluorescence confocal microscopy demonstrated cytoplasmic co-localization of YBX1 and LDHA (Fig. 5D). Next, molecular docking analysis predicted a binding interaction between the two proteins (Fig. 5E). To identify the key YBX1 domains mediating this interaction, we transfected 786-O cells with GFP-tagged truncated YBX1 constructs (Fig. 5F). GFP pull-down assays revealed that LDHA interacts with full-length YBX1 (FL), the N-terminal region (1–129 aa), and the linker region (130-205 aa) (Fig. 5G). These findings align with molecular docking results, which localized the binding site to 51–205 aa. Notably, the cold-shock domain (CSD, 51-129 aa) primarily mediates DNA/RNA binding, while the C-terminal domain (CTD, 130–324 aa) facilitates protein-protein interactions. This structural insight suggests that the transcription factor YBX1 may directly bind to the LDHA gene promoter, providing a potential mechanism for its regulatory role. Next, we utilized a set of Flag-tagged LDHA truncated variants (Fig. 5H). We transfected plasmids containing these truncated variants into 786-O cells and performed pull-down assays using the Flag tag. The results of the immunoprecipitation demonstrated that the LDHA mutant lacking the 21–163 aa region does not interact with YBX1, indicating that this region is essential for the interaction between LDHA and YBX1 (Fig. 5I). We conducted experiments by transfecting plasmids containing YBX1 domain constructs to assess LDH activity. Our results indicated that transfection with YBX1 domain plasmids that interact with LDHA (FL, 1–129 aa, and 130–205 aa) significantly enhanced LDH activity, whereas transfection with the YBX1 domain plasmid that does not interact with LDHA (206–324 aa) did not promote LDH activity (Fig. 5J). This finding demonstrates that the interaction between YBX1 and the LDHA protein influences its activity.

### YBX1 regulates LDHA transcriptional activation

**Fig. 6:**
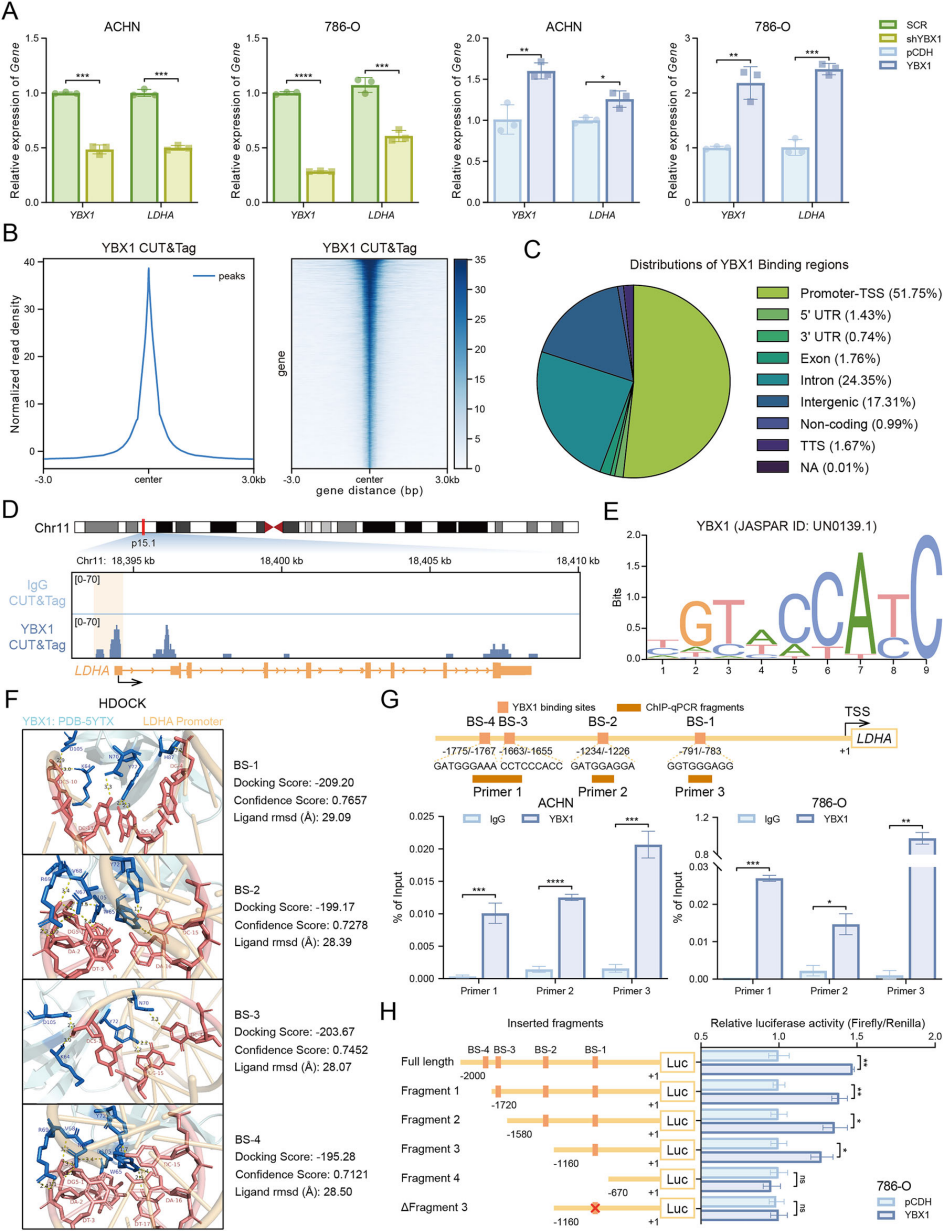
YBX1 regulates LDHA transcriptional activation. **A** qRT-PCR analysis of *LDHA* mRNA expression levels in ACHN and 786-O cells following YBX1 knockdown and overexpression. **B** CUT&Tag peak plots and heatmap showing YBX1 binding intensity in ACHN cells. **C** Pie chart showing the distribution of YBX1 binding regions. **D** IGV plot showing the occupancy of YBX1 in the *LDHA* promoter region. **E** Motif diagram of YBX1 binding sequences from the JASPAR database. **F** Molecular docking analysis of YBX1 with four predicted binding sites (BS) in the *LDHA* promoter sequence. **G** The *LDHA* promoter sequence was divided into 3 fragments containing the predicted BS, followed by primer design and ChIP-qPCR to verify YBX1 binding to the *LDHA* promoter region. **H** The LDHA promoter sequence was divided into 4 fragments, and the BS-1 site that was mainly bound was mutated, the luciferase-tagged truncated plasmids were designed. After co-transfection of the target and control Renilla plasmids into ccRCC cells, YBX1 binding to the LDHA promoter region was verified using dual-luciferase reporter assays. *P < 0.05, **P < 0.01, ***P < 0.001, ****P < 0.0001, ns: no significant difference. Unpaired two-sided Student’s *t*-test in A, G and H. Data are presented as mean ± SD.

To investigate the potential relationship between YBX1 and LDHA at the transcriptional level, we performed qRT-PCR to assess *LDHA* expression following YBX1 knockdown and overexpression in ACHN and 786-O cells. The results showed that LDHA expression was significantly decreased in YBX1-knockdown ccRCC cells compared to controls, while it was significantly increased in YBX1-overexpressing cells (Fig. 6A). YBX1 functions as a transcription factor to regulate gene expression and can also modulate DNA repair, RNA splicing, exon skipping, drug resistance, and cancer progression through mechanisms independent of nucleic acid binding motifs [9]. To identify YBX1 binding sites genome-wide, we performed CUT&Tag assays using anti-YBX1 antibodies, detecting 14,295 peaks compared to IgG control signals (Fig. 6B). Analysis of YBX1 binding site distribution revealed that approximately 51.75% were enriched in promoter and transcription start site (TSS) regions (Fig. 6C). Notably, we observed significant YBX1 enrichment in the *LDHA* promoter region (Fig. 6D). Using the JASPAR database, we identified the YBX1 binding motif (Fig. 6E) and analyzed the *LDHA* promoter region (±2000 bp from TSS), predicting four potential binding sites. Molecular docking indicated YBX1 interacts with all four *LDHA* promoter segments, with BS-1 showing the highest docking score (-209.20) and confidence level (0.7657) (Fig. 6F). To validate these findings, we constructed four truncated reporter plasmids containing each binding site. Dual-luciferase assays demonstrated that YBX1 overexpression specifically enhanced transcriptional activity from BS-1 (-1160 to -670 bp), confirming this region as the primary YBX1 binding site in the *LDHA* promoter. Subsequently, we mutated the BS-1 site and found that the activation of *LDHA* by YBX1 was inhibited, indicating that the BS-1 site is responsible for the regulation of *LDHA* by YBX1 (Fig. 6H). These results demonstrate that YBX1 transcriptionally activates *LDHA* by directly binding to its promoter region.

### YBX1/LDHA activates the NF-κB signaling pathway to promote ccRCC glycolysis

**Fig. 7:**
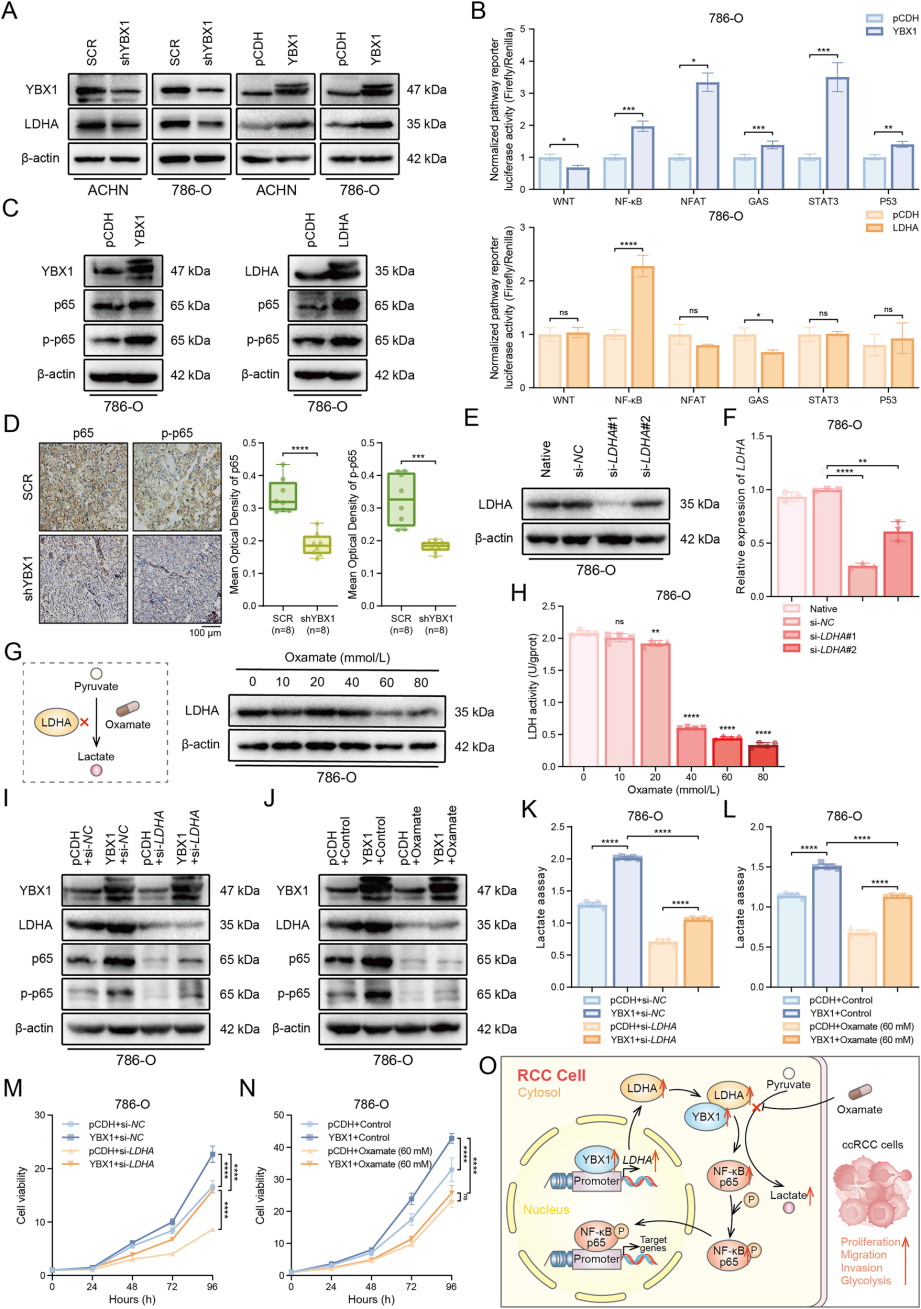
YBX1/LDHA promotes glycolysis by activating the NF-κB pathway. **A** Western blot analysis of LDHA protein expression levels in ACHN and 786-O cells after YBX1 knockdown and overexpression. **B** Dual-luciferase reporter assays evaluating the effects of YBX1 and LDHA on glycolysis-related signaling pathways in ccRCC cells co-transfected with target plasmids and control Renilla plasmids. **C** Western blot analysis of p-p65 (Ser536) and total p65 expression after overexpression of YBX1 and LDHA. **D** Immunohistochemical staining to detect protein expression of total p65 and p-p65 (Ser536) in tumor tissues from control and YBX1 knockdown groups in nude mice. Representative images and quantification. **E, F** Western blot (**E**) and qRT-PCR (**F**) analysis of LDHA knockdown efficiency in 786-O cells 72 h post-transfection with si-*LDHA*. **G**, **H** Western blot (**G**) and LDH activity (**H**) analysis of LDHA inhibition in 786-O cells treated with Oxamate (0-80 mM for 48 h). **I**, **J** Western blot analysis of p-p65 (Ser536) and total p65 in YBX1-overexpressing cells following LDHA knockdown (**I**) or Oxamate treatment (60 mM for 48 h) (**J**). **K-N** Lactate production (**K, L**) and cell proliferation (**M**, **N**) assays in YBX1-overexpressing cells after LDHA knockdown or Oxamate treatment. **O** Schematic diagram illustrating the YBX1-LDHA-NF-κB mechanism in ccRCC progression. *P < 0.05, **P < 0.01, ***P < 0.001, ****P < 0.0001, ns: no significant difference. Unpaired two-sided Student’s *t*-test in **B**, **D**, **F** and **H**. One-way ANOVA with correction for multiple comparisons in K and L. Two-way ANOVA with correction for multiple comparisons in M and N. Data are presented as mean ± SD.

Our results demonstrate that YBX1 not only binds to and activates LDHA at the transcriptional level but also associates with LDHA at the protein level, revealing a positive correlation between the two. Western blot analysis confirmed that YBX1 regulates LDHA expression at the protein level (Fig. 7A). To investigate the specific mechanism by which YBX1 influences ccRCC glycolysis via LDHA, we first used a dual-luciferase reporter assay to screen potential signaling pathways that might be affected by the overexpression of YBX1 and LDHA, including the WNT, NF-κB, NFAT, GAS, STAT3, and P53 pathways. Literature research suggested that these pathways may regulate glycolysis. The results showed that overexpression of YBX1 and LDHA jointly activated the NF-κB signaling pathway (Fig. 7B), consistent with previous reports that LDHA induces NF-κB pathway activation [34]. We then performed western blot analysis to assess the effects of YBX1 and LDHA on the NF-κB subunit p65 and phosphorylated p65 (Ser536). The results showed that overexpression of YBX1 and LDHA upregulated the protein levels of both p65 and p-p65 (Fig. 7C). We detected p65 and p-p65 in nude mice tumor tissue sections by immunohistochemical staining, and the results showed that the protein expression of p65 and p-p65 was also significantly reduced after YBX1 was down-expressed (Fig. 7D). To further confirm whether YBX1 regulates the NF-κB signaling pathway through LDHA in ccRCC cells, we transfected 786-O cells with small interfering RNA (siRNA) targeting LDHA (si-*LDHA*). Western blot and qRT-PCR results confirmed a significant reduction in both protein and mRNA levels of LDHA (Fig. 7E, F). We also used the LDHA inhibitor Oxamate to test the inhibitory effects and activity on LDHA at various concentrations in 786-O cells (Fig. 7G, H). Subsequently, in both YBX1-overexpressing and control cells, we transfected si-*LDHA* and si-*NC*, and assessed the impact of YBX1-LDHA regulation on the NF-κB signaling pathway through western blot analysis. The results revealed that overexpression of YBX1 increased the protein levels of p65 and p-p65, while LDHA downregulation diminished the ability of YBX1 to activate the NF-κB signaling pathway (Fig. 7I). Similarly, treatment with Oxamate in YBX1-overexpressing cells yielded the same results (Fig. 7J). Therefore, YBX1 regulates the NF-κB signaling pathway through LDHA. To examine whether this interference and inhibition subsequently affect ccRCC glycolysis and progression, we conducted lactate production and cell proliferation assays. As expected, LDHA depletion significantly reversed the increase in lactate production (Fig. 7K, L) and proliferation (Fig. 7M, N) caused by YBX1 overexpression in 786-O cells. These results indicate that YBX1 regulates glycolysis and cell proliferation in ccRCC cells through LDHA (Fig. 7O).

## Discussion

RCC is the most common malignant tumor of the urinary system, and its incidence has been steadily rising in recent years [35]. Most RCC cases are asymptomatic, making them difficult to diagnose in the early stages and resulting in poor prognoses [36]. Currently, the main treatments for RCC include surgery, radiotherapy, as well as targeted and non-targeted therapies [37]. Since RCC is not sensitive to radiotherapy, gene-targeted therapy provides a promising new direction for RCC treatment [38]. RCC is sometimes characterized as a metabolic disease, and almost all types of kidney cancer are related to tumor metabolic reprogramming [39]. The Warburg effect is considered a hallmark of cancer and plays a critical role in cancer development [40]. Compared with normal cells, cancer cells exhibit a higher propensity for glycolysis, regardless of whether they are in normoxic or hypoxic environments [41]. YBX1 is an oncogenic transcription factor that is overexpressed in many cancer cells, including RCC, and is associated with tumor growth, angiogenesis and metastasis [42]. CIRC-SAR1A upregulates YBX1 expression by sponging miR-382, thereby promoting the growth and invasion of RCC cells [43]. In triple-negative breast cancer (TNBC), knockdown of YBX1 inhibited the expression of glycolytic genes (*ENO1*, *SLC2A6*, *LDHA*, *PFKP*, *PGAM1*, and *GPI*) and downregulated the expression of EMT-related genes and tumor migration and invasion [14]. YBX1 regulates the expression of c-Myc and HIF1α in bladder cancer, further upregulating glycolytic enzymes to promote glycolysis [15], but the role of YBX1 in regulating glycolysis in kidney cancer has not been reported. In this study, we found that YBX1 is an oncogene in RCC, which is related to RCC glycolysis and cell proliferation, indicating that YBX1 may serve as a potential marker of RCC.

To investigate the mechanism of YBX1 in aerobic glycolysis, we identified LDHA through IP-MS. LDHA is an important rate-limiting enzyme in the glycolytic pathway, playing a crucial role in promoting tumor malignancy by regulating tumor energy metabolism [44]. LDHA is overexpressed in many cancer cells, including kidney cancer [19], breast cancer [45], and colorectal cancer [46], and promotes cancer development and progression. Through bioinformatics analysis and clinical sample studies, we found that both YBX1 and LDHA are highly expressed in RCC and positively correlated with each other. Additionally, YBX1 can interact with LDHA, thereby influencing LDH activity. Considering the important role of YBX1 and LDHA expression in RCC glycolysis, we sought to determine the potential mechanism of YBX1 and LDHA co-expression in RCC. We investigated the effect of altered YBX1 expression on LDHA and found that YBX1 overexpression leads to increased LDHA expression, while decreased YBX1 expression has the opposite effect. It has been demonstrated that *LDHA* is a downstream target gene of HIF [47], c-myc [48], and HOXC4 [49]. Since YBX1 is a transcription factor, we further studied YBX1 and determined that it can regulate LDHA expression through transcriptional mechanisms. We found that YBX1 directly binds to the promoter region of LDHA and regulates its promoter activity. These data suggest that YBX1 transcriptionally regulates LDHA expression.

Nuclear factor κB (NF-κB) is a key pathway regulating various cellular functions, including proliferation, apoptosis and immune response [50]. In cholangiocarcinoma, KIF14 binds to the G3BP1/YBX1 complex, leading to the activation of the NF-κB pathway [51]. It has been demonstrated that YBX1 methylation mediated by PRMT5 can regulate NF-κB activity in colorectal cancer [52]. Additionally, phosphorylation of YBX1 can activate the NF-κB pathway in colon cancer [53, 54]. Moreover, YBX1 enhances cisplatin sensitivity in neuroblastoma through this pathway [55]. Research has shown that NF-κB dysregulation impedes RCC progression and contributes to drug resistance [21]. Our research group previously demonstrated that YBX1 interacts with G3BP1, which promotes RCC cells migration and invasion by activating the SPP1/NF-κB signaling pathway [13]. Similarly, Li et al. found that LDHA and its main metabolite lactate drive nasopharyngeal carcinoma progression by regulating TAK1 and its downstream NF-κB signaling [56]. It has been reported that miR-16-5p can inhibit LDHA, leading to decreased lactate accumulation and aerobic glycolysis, thereby inhibiting NF-κB activation [57]. NF-κB can directly activate the expression of glycolysis-related genes, such as glucose transporter (GLUT) [58], or by activating the PI3K/Akt signaling pathway, which in turn can phosphorylate and activate PFKFB3 to promote glycolysis [59]. Under hypoxic conditions, NF-κB can interact with HIF-1α to enhance the stability of HIF-1α [60], which activates a series of genes related to glycolysis, thereby promoting glycolysis. Furthermore, OLFM4 regulates the pro-inflammatory response of lung epithelial cells in sepsis-related ARDS through LDHA-mediated activation of the NF-κB signaling pathway [61]. Based on these findings, we evaluated the effects of YBX1 and LDHA on NF-κB signaling in RCC cells. We found that YBX1 and LDHA can affect the expression of p65 protein and the phosphorylation level of p65 (Ser536) in RCC cells. Moreover, we proved that si-*LDHA* and Oxamate, an LDHA inhibitor, reversed the YBX1-mediated enhancement of NF-κB activation. Considering the role of NF-κB in promoting tumors and regulating energy metabolism in cancer [62, 63], we further demonstrated the function of YBX1 in glycolysis and RCC progression mediated by LDHA through lactic acid production and CCK-8 proliferation assays.

In summary, this study demonstrated that YBX1 participates in RCC metabolism and promotes RCC cell proliferation, invasion and metastasis. YBX1 and LDHA are highly expressed in RCC and positively correlated. Notably, YBX1 interacts with LDHA and enhances its transcription. In addition, YBX1 can activate the NF-κB pathway through LDHA. Therefore, the YBX1-LDHA-NF-κB signaling axis may represent a potential therapeutic target for RCC.

## Supplementary information

Supplementary table

Uncropped Western Blots

qPCR data

## Data Availability

The datasets used and/or analyzed during the current study are available from the corresponding author on reasonable request.
