# Supplementary material for: YBX1 orchestrates LDHA-mediated metabolic reprogramming and NF-κB activation to drive clear cell renal cell carcinoma progression
Source: Cell Death Dis. 2026 Jan 8;17(1):11. doi: 10.1038/s41419-025-08261-0 (PMC12783105; doi:10.1038/s41419-025-08261-0)
Supplement: Supplementary file 1 — Supplementary table [file 41419_2025_8261_MOESM1_ESM.docx]

**Supplementary Table 1.** Targeted sequences of siRNAs in this study.

| siRNA | Sequences |
| --- | --- |
| si-*LDHA*#1 | GCUACACAUCCUGGGCUAUTT |
| si-*LDHA*#2 | AGGTTCACAAGCAGGTGG |

**Supplementary Table 2**. Antibodies used in this study.

| Antibodys | Manufacturers | Application |
| --- | --- | --- |
| YBX1 | ab76149, Abcam, UK | WB 1:2000, IHC 1:50, Co-IP 4 μg |
| YBX1 | 20339-1-AP, Proteintech, USA | WB 1:2000, CUT&Tag 1 μg, ChIP 4 μg |
| YBX1 | sc-398340, Santa Cruz Biotechnology, USA | WB 1:500, IF 1:50 |
| LDHA | ab101562, Abcam, UK | WB 1:2000, IHC 1:50, IF 1:50, Co-IP 4 μg |
| LDHA | 19987-1-AP, Proteintech, USA | WB 1:3000 |
| GFP | G1544, SIGMA, Germany | Co-IP 4 μg |
| GFP | T0005, Affinity, USA | WB 1:3000 |
| Flag | 8146S, Cell Signaling Technology, USA | WB 1:2000 |
| p65 | AF5006, Affinity, USA | WB 1:1000, IHC 1:50 |
| p-p65 | AF2006, Affinity, USA | WB 1:2000, IHC 1:50 |
| β-actin | T0022, Affinity, USA | WB 1:3000 |
| HRP-conjugated goat anti-Rabbit IgG | S0001, Affinity, USA | WB 1:3000 |
| HRP-conjugated goat anti-Mouse IgG | S0002, Affinity, USA | WB 1:3000 |
| Ki-67 | ab15580, Abcam, UK | IHC 1:500 |
| Alexa-Fluor 488 AffiniPure Goat Anti-Rabbit IgG | A23220, Abbkine, China | IF 1:100 |
| Alexa-Fluor 594 AffiniPure Goat Anti-Mouse IgG | A23410, Abbkine, China | IF 1:100 |
| DAPI | C0060, Solarbio, China | IF 1:100 |
| IgG | 2729S, Cell Signaling Technology, USA | Co-IP 4 μg, CUT&Tag 1 μg, ChIP 4 μg |

**Supplementary Table 3.** The qRT-PCR primers used in this study.

| Gene names | Primers Sequences |
| --- | --- |
| *YBX1* | Forward: GGGTGCAGGAGAACAAGGTA |
|  | Reverse: TCTTCATTGCCGTCCTCTCT |
| *LDHA* | Forward: GAGTGGAATGAATGTTGCTGGTGTC |
|  | Reverse: CCAGGATGTGTAGCCTTTGAGTTTG |
| *GAPDH* | Forward: GCCGTCTATGCGGCTTGT |
|  | Reverse: TGGAAGGGGTTCCCTGAGTT |

**Supplementary Table 4.** The ChIP-qPCR primers used in this study

| Gene names | Primers Sequences |
| --- | --- |
| Primer 1 | Forward: AAAAATCGCAGGGCAAGTGG |
|  | Reverse: CGTGATCATGGCTTCCTGCA |
| Primer 2 | Forward: CCACAGTGGGGAAGTATTGGT |
|  | Reverse: TTATCATACTTACCTCTGGGCCTG |
| Primer 3 | Forward: TGTGTTTGAGGTGAGAGCCC |
|  | Reverse: ATGCCTGTAATCCCAGCACC |
