# Supplementary figures and images for: YBX1 orchestrates LDHA-mediated metabolic reprogramming and NF-κB activation to drive clear cell renal cell carcinoma progression
Source: Cell Death Dis. 2026 Jan 8;17(1):11. doi: 10.1038/s41419-025-08261-0 (PMC12783105; doi:10.1038/s41419-025-08261-0)

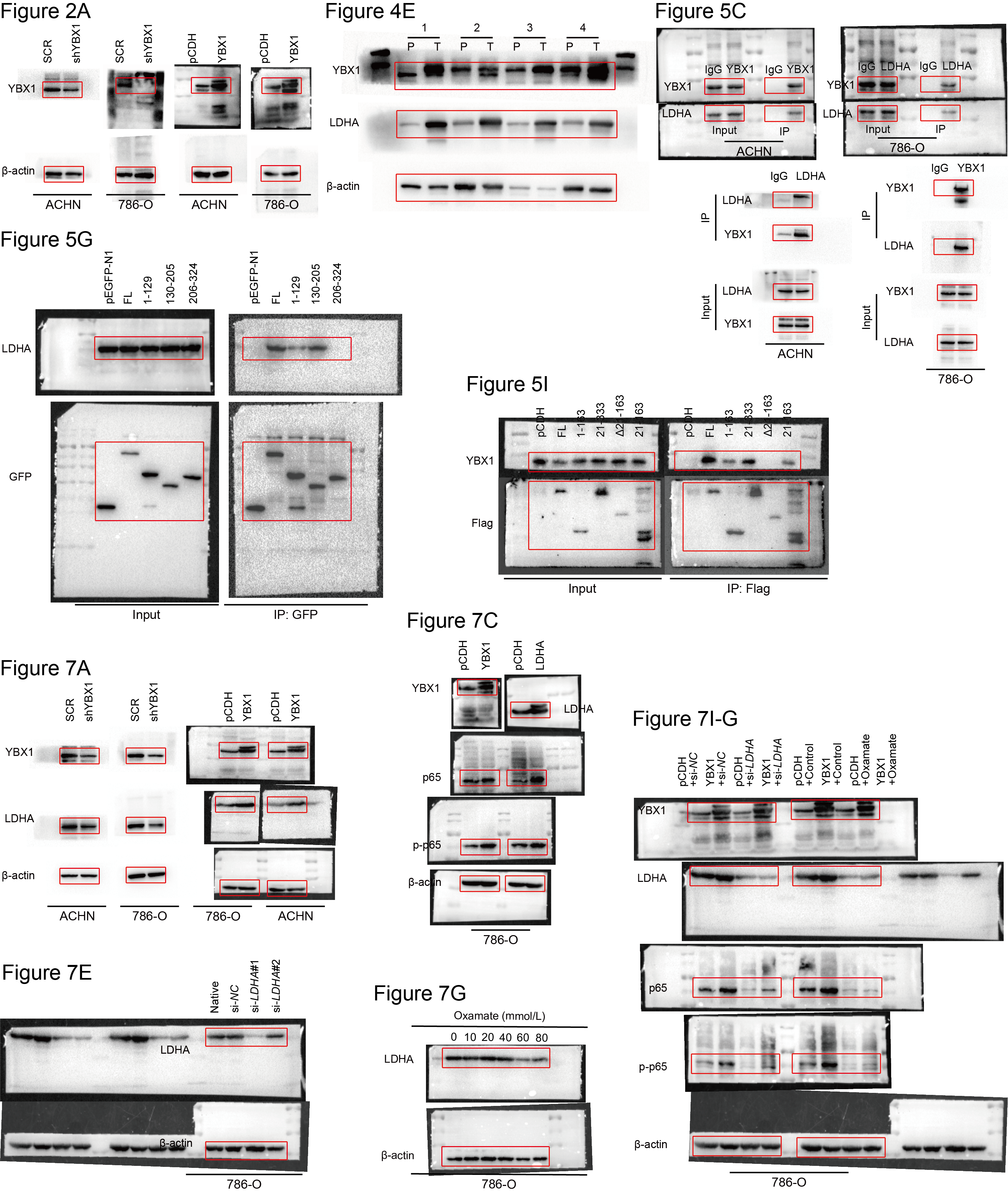

Supplement: Supplementary file 2 — Uncropped Western Blots [file 41419_2025_8261_MOESM2_ESM.png]
